# Trends in pediatric firearm-related injuries and disparities in acute outcomes

**DOI:** 10.3389/fpubh.2024.1339394

**Published:** 2024-03-19

**Authors:** Makda G. Mulugeta, Gabrielle Bailey, Kendall Parsons, Scott Gillespie, Laura M. Johnson, Kiesha Fraser Doh, Andrew Reisner, Laura S. Blackwell

**Affiliations:** ^1^Children's Healthcare of Atlanta, Atlanta, GA, United States; ^2^Department of Pediatrics, Emory University School of Medicine, Atlanta, GA, United States; ^3^Department of Neurosurgery, Emory University School of Medicine, Atlanta, GA, United States

**Keywords:** pediatric, firearm, COVID-19, health disparity, social determinants of health, firearm-related injuries

## Abstract

**Background:**

Firearm-related injuries (FRI) are an increasing cause of death and injury in children. The etiology for this rise is multifactorial and includes socioeconomic factors. Despite its prevalence and documented increase over COVID-19, there is a paucity of research on disparities and the influence of social determinants of health (SDH) in pediatric FRI. This study aims to explore the epidemiology of this vulnerable population in Atlanta, trends over time and relevant dates such as COVID-19 and a state firearm law, and disparities in clinical outcomes.

**Methods:**

Retrospective cohort of patients with FRI (0–20 years-old, x̄=9.8, Median = 11) presenting to our hospital EDs from January 2014 to April 2023 (*N* = 701) and eligible for the Trauma Registry. This period includes two major events, namely the COVID-19 pandemic (March 2020), and passage of state law Constitutional Carry Act (SB 319) (April 2022), allowing for permit-less concealed firearm carry. Single series interrupted time series (ITS) models were run and clinical outcome differences between race and insurance groups were calculated unadjusted and adjusted for confounders using inverse propensity treatment weights (IPTW). The primary outcome was mortality; secondary are admission and discharge.

**Results:**

Majority of FRI involved patients who were male (76.7%), Black (74.9%), publicly insured (82.6%), ≤12 years-old (61.8%), and injured by unintentional shootings (45.6%) or assault (43.7%). During COVID-19, there was a sustained increase in FRI rate by 0.42 patients per 1,000 trauma visits per month (95% CI 0.02–0.82, *p* = 0.042); post-SB 319 it was 2.3 patients per 1,000 trauma visits per month (95% CI 0.23–4.31, *p* = 0.029). Publicly insured patients had 58% lower odds of mortality than privately insured patients (OR 0.42, 95% CI 0.18–0.99, *p* = 0.047). When controlled for race and mechanism of injury, among other confounding factors, this association was not significant (*p* = 0.652).

**Conclusion:**

Pediatric FRI are increasing over time, with disproportionate burdens on Black patients, at our hospitals. Disparities in mortality based on insurance necessitate further study. As social and economic repercussions of COVID-19 are still present, and state firearm law SB 319 is still in effect, assessment of ongoing trends is warranted to inform preventative strategies.

## 1 Introduction

Firearm-related injuries (FRI) are an increasing cause of morbidity and mortality among children and adolescents in the United States (U.S.). More than 3,900 children and adolescents die from FRI annually, and thousands more surviving children and adolescents are left with varying degrees of both physical and emotional injuries (1–3). Children and adolescents in the U.S. are estimated to be 36.5 times more likely to die from FRI compared to similar-aged children in other high-income countries (4).

In recent years, FRI surpassed motor vehicle collisions (MVCs) and became the leading cause of pediatric mortality in the U.S. (5, 6). Although pediatric trauma rates remained the same or even decreased, pediatric FRI continued to increase during the COVID-19 pandemic (7, 8). To further complicate this narrative, one study found that nonfatal *per capita* injuries in pediatrics have been decreasing while fatal injuries have been increasing in recent years (3). Taken together, these studies may indicate that while overall trauma rates have been decreasing over time, a greater proportion of injuries may be related to FRI. In 2021, 4,752 children and adolescents died from FRI, which translates to an average of 13 children every day (1). While it is likely that the COVID-19 pandemic contributed to this increase with economic instability, school closures, and social isolation (9, 10), additional factors such as state firearm regulation laws (11) and record setting firearm purchases (12, 13) also likely contributed. Recently in Georgia, a law passed, SB 319, that allowed for concealed carry of firearms without a permit (14). Both COVID-19 and this firearm law relate to and potentially impact pediatric FRI in our hospital system located in Atlanta, Georgia.

There is a paucity of literature that focus on populations most at risk. Regarding demographic groups, males, older adolescents, and children from minoritized groups (15) are known to have higher rates of FRI (1, 16). Black children are over 70% more likely to be hospitalized for FRI than White children; Hispanic children are 20% more likely to be hospitalized than White children (17). Regardless of neighborhood income level, Black children still have higher rates of FRI compared to White children (17). Within the U.S., Southern states have a disproportionately high volume of pediatric firearm incidents (18, 19).

Social determinants of health (SDH), the non-medical, environmental, and social conditions we live in, drive health inequalities in disease and injury (20) and FRI is no exception. Recent studies have shown that neighborhood poverty and deprivation associates with increased pediatric FRI risk and mortality (1, 19, 21, 22). Additionally, prior studies have shown that insurance associates with mortality following FRI (23) and in pediatric trauma (24, 25). Although these studies are few in number, they point to an urgent need to address the impact of SDH on pediatric FRI. As injuries are preventable, identifying trends in FRI in relation to SDH can inform preventative strategies (26).

The aims of this study are: (1) to characterize the trends in pediatric FRI with respect to patient demographics prior to and during the COVID-19 pandemic in our exclusive pediatric hospital system; (2) to examine the incidence of these injuries with respect to COVID-19 and a recent Georgia firearm law; (3) examine how SDH (race and insurance) relate to acute outcomes following FRI.

## 2 Materials and methods

### 2.1 Procedures

This study is a retrospective analysis using a hospital-based trauma registry of pediatric patients who presented to Children’s Healthcare of Atlanta’s (CHOA) Level I or Level II pediatric trauma centers between January 2014 and April 2023 with FRI as identified by the International Classification of Diseases (ICD) 9th and 10th revision codes. CHOA has the only pediatric trauma centers in Georgia’s capital, Atlanta, and are two of three pediatric trauma centers in the state (27). Thus, our patient population covers a large portion of the state. Both ICD-9 and 10 codes were included as the transition from ICD-9 to ICD-10 took place on July 2015, during the study timeline. CHOA’s Institutional Review Board approved this study.

The hospital-based trauma registry included patients who qualify for the National Trauma Data Standards (NTDS). Inclusion criteria for this registry included patients who sustained a traumatic injury within 2 weeks of their first hospital encounter, had at least one ICD-9 or ICD-10 code identifying traumatic injury, and either died as a result of injury in the Emergency Department (ED), were transferred from one acute care hospital to another via Emergency Medical Services (air or ground transportation), or were admitted to one of our two pediatric trauma centers (Scottish Rite and Egleston). Exclusion criteria in the NTDS included specific codes (such as superficial injury), traumatic injuries that occurred during the hospital encounter, and admissions for elective or planned surgeries. In addition to the NTDS, the hospital-based trauma registry included all trauma-related deaths and patients with trauma-related mechanisms of injury who were admitted or transferred to/from another acute care hospital.

Patients with ICD codes indicating injury from firearms (e.g., handgun, rifle, shotgun, etc.), gas, air, or spring-operated guns, were included in the study. Non-powder guns have been included due to their capacity to cause significant injury in children (28, 29). From the patients that fit the aforementioned criteria, the study dataset was formed using the following trauma registry variables: demographics (age, race, ethnicity, and gender), injury circumstance (mechanism of injury), acute outcomes (discharge from the Emergency Department [ED], hospital discharge destination, mortality, and Injury Severity Score [ISS]), and insurance. Ethnicity and race were recorded in the trauma registry as separate variables, and thus will be reported in this manner. Mechanism of injury was classified into assault, unintentional, intentional self-infliction, undetermined (unable to determine intent due to lack of information), legal intervention (police/law enforcement shooting), child abuse, or other based on keywords from ICD External Cause Codes (E-Codes) (30).

Patients were categorized by age groups identified in prior literature to reflect developmental subgroups (31–34). Age groups were as follows; 0–6 years, 7–12 years, 13–15 years, and 16–20 years. We chose to separate the adolescent age group into younger (13–15 years) and older (16–20 years) categories due to the varying mechanisms of FRI that have been shown to impact these age groups within the literature (35). The sample did not have any patients aged 21 years, despite eligibility. Discharge from ED was categorized into admitted to hospital (floor, Intensive Care Unit [ICU], Operative Room [OR], or direct admit to hospital), discharged home, died, or discharged to another hospital. Hospital discharge destination was categorized into home, another hospital/intermediate care facility (e.g., psychiatric hospital, short-term general hospital, etc.), died, N/A (patients who were never admitted into the hospital), court/law enforcement, and inpatient rehabilitation. Insurance was classified into private (government or private/commercial), public (Medicaid or Medicaid type insurances), and uninsured (self-pay). Insurance and race serve as the SDH for this study based on the available data within our dataset. For contextual analysis, a dataset of all patients in the trauma registry over the study period (January 2014 to April 2023) was formed with variables including hospital admission date and race.

To examine changes in rates of FRI during the COVID-19 pandemic, this study utilized a cut-off date of March 13th, 2020, the official U.S. emergency declaration date (36). This study examines a recent state firearm law, the Constitutional Carry Act (SB 319), to assess FRI trends in relation to firearm laws (14). Effective on April 12th, 2022, this law allowed for the concealed carry of firearms without a permit (14, 37, 38).

### 2.2 Statistical analyses

All analyses were performed in SAS v.9.4 (Cary, NC) and CRAN R v.4.3 (Vienna, Austria), and statistical significance was evaluated throughout at the 0.05 threshold. As aforementioned, patients were grouped by race and ethnicity separately based on the database. First, single series interrupted time series (ITS) models were run to evaluate the rate of FRI per 1,000 trauma visits over 9 years of data. Overall trauma visits were used as the comparison to differentiate FRI and general trauma rates over time, assuming that general trauma numbers at our hospital may vary year to year. Then, clinical outcome (e.g., acute outcome) differences between Black and White races for patients with FRI were calculated unadjusted and adjusted for confounders using inverse propensity treatment weights (IPTW). Steps for ITS included statistical tests and visual inspection for autocorrelation and seasonality, and regression results were based on piecewise linear equations (intercepts and slopes) calculated pre- and post-interruptions for COVID-19 era (March 2020) and Constitutional Carry Act era (April 2022). Two individual and one combined time series were performed. A combined time series analysis was performed to assess for the combined effects of both events.

For the clinical outcomes analysis, which considered binary outcomes, Firth logistic regression was employed and reported by odds ratios with 95% confidence intervals (CI) and *p*-values. Firth’s Penalized Likelihood was utilized to account for the bias that can occur with rare outcomes. All outcomes analysis, demographic, SDH, and clinical differences between Black and White patients and insurance groups, were balanced using IPTW derived from the *twang* v.2.5 package in CRAN R. Specifically, average treatment effect (ATE) weights were calculated with a gradient boosted model (GBM) using 10,000 trees, interaction depth at 3, and a stop method based on mean effect size. Final IPTW were stabilized to approximately match the original study sample size and trimmed at the 1 and 99%. Checking of confounders with IPTW demonstrated balance when standardized mean differences (SMD) <0.25. All outcome analyses between the patient groups by race and insurance status were weighted with IPTW. Insurance was only examined with mortality due to insurance’s effect on patient hospital course (secondary outcomes) (39).

## 3 Results

### 3.1 Demographic characteristics

In total, 701 children and adolescents were treated for FRI at our hospitals between January 2014 and April 2023. Annual number of patients increased 381% over the 9-year study period (*n* = 27 in 2014 vs. *n* = 130 in 2022) (Table 1). The cohort was predominantly male (76.7%), Black (74.9%), Non-Hispanic/Latino (93.2%), and publicly insured (82.6%). Patient ages range from 0 to 20 years. The largest age group was 7–12 year-olds (33.5%), followed by 13–15 year-olds (29.5%), 0–6 year-olds (28.3%), and 16–20 year-olds (8.7%) (average = 9.8, median = 11). Over 2020, Black patients with FRI increased (63.2% in 2019, 75.5% in 2020, 83.9% in 2021) while White patients with FRI decreased (31.6% in 2019, 17.6% in 2020, 11% in 2021). Over time, patients were increasingly publicly insured (70.4% in 2014, 93.1% in 2022), and decreasingly privately insured (25.9% in 2014, 6.1% in 2022). The most common mechanisms of injury were unintentional shooting (45.6%) and assault (43.7%). As of April, 65.6% of all patients in 2023 were injured by assault – twice the proportion of patients in 2014 (33.3%).

**Table 1:** Participant characteristics by year (*N* = 701).

Characteristic, *N* (col. %)	2014 *N* = 27	2015 *N* = 38	2016 *N* = 30	2017 *N* = 61	2018 *N* = 56	2019 *N* = 77	2020 *N* = 103	2021 *N* = 118	2022 *N* = 130	2023 *N* = 61
Sex
Female	6 (22.2%)	12 (31.6%)	7 (23.3%)	19 (31.1%)	14 (25%)	15 (19.5%)	29 (28.2%)	24 (20.3%)	25 (19.2%)	12 (19.7%)
Male	21 (77.8%)	26 (68.4%)	23 (76.7%)	42 (68.9%)	42 (75%)	62 (80.5%)	74 (71.8%)	94 (79.7%)	105 (80.8%)	49 (80.3%)
Race, *N* = 694
Black	19 (70.4%)	27 (71.1%)	20 (69%)	46 (75.4%)	40 (71.4%)	48 (63.2%)	77 (75.5%)	99 (83.9%)	99 (78.6%)	50 (82%)
White	8 (29.6%)	10 (26.3%)	5 (17.2%)	14 (23%)	11 (19.7%)	24 (31.6%)	18 (17.6%)	13 (11%)	20 (15.9%)	8 (13.1%)
Mixed/Other Race	0 (0%)	1 (2.6%)	4 (13.8%)	1 (1.6%)	5 (8.9%)	4 (5.2%)	7 (6.9%)	6 (5.1%)	7 (5.5%)	3 (4.9%)
Unknown	0	0	1	0	0	1	1	0	4	0
Ethnicity, *N* = 700
Hispanic/Latino	0 (0%)	3 (7.9%)	2 (6.7%)	3 (4.9%)	3 (5.4%)	7 (9.1%)	7 (6.8%)	7 (5.9%)	9 (7%)	6 (9.8%)
Non-Hispanic/Latino	27 (100%)	35 (92.1%)	28 (93.3%)	58 (95.1%)	53 (94.6%)	70 (90.9%)	96 (93.2%)	111 (94.1%)	120 (93%)	55 (90.2%)
Unknown	0	0	0	0	0	0	0	0	1	0
Age
0–6	8 (29.6%)	10 (26.3%)	10 (33.3%)	13 (21.3%)	15 (26.8%)	22 (28.6%)	24 (23.3%)	42 (35.6%)	37 (28.5%)	17 (27.9%)
7–12	10 (37%)	16 (42.1%)	14 (46.7%)	24 (39.3%)	20 (35.7%)	29 (37.6%)	26 (25.2%)	30 (25.4%)	46 (35.4%)	20 (32.8%)
13–15	6 (22.2%)	10 (26.3%)	4 (13.3%)	22 (36.1%)	19 (33.9%)	26 (33.8%)	42 (40.8%)	31 (26.3%)	34 (26.1%)	13 (21.3%)
16–20	3 (11.2%)	2 (5.3%)	2 (6.7%)	2 (3.3%)	2 (3.6%)	0 (0%)	11 (10.7%)	15 (12.7%)	13 (10%)	11 (18%)
Insurance, *N* = 697
Private	7 (25.9%)	4 (10.8%)	2 (6.7%)	8 (13.1%)	5 (8.9%)	10 (13%)	12 (11.8%)	14 (12.1%)	8 (6.1%)	0 (0%)
Public	19 (70.4%)	30 (81.1%)	24 (80%)	47 (77.1%)	40 (71.3%)	54 (70.1%)	82 (80.4%)	102 (87.9%)	121 (93.1%)	60 (98.4%)
Uninsured	1 (3.7%)	3 (8.1%)	4 (13.3%)	6 (9.8%)	11 (19.6%)	13 (16.9%)	8 (7.8%)	0 (0%)	1 (0.8%)	1 (1.6%)
Unknown	0	1	0	0	0	0	1	2	0	0
Mechanism of injury
Unintentional	15 (55.6%)	18 (47.4%)	15 (50%)	34 (55.7%)	27 (48.2%)	44 (57.1%)	43 (41.7%)	47 (39.8%)	60 (46.2%)	17 (27.9%)
Assault	9 (33.3%)	11 (28.9%)	9 (30%)	19 (31.1%)	25 (44.6%)	28 (36.4%)	50 (48.5%)	51 (43.2%)	64 (49.2%)	40 (65.6%)
Child Abuse/Legal Intervention	0 (0%)	0 (0%)	0 (0%)	0 (0%)	1 (1.8%)	0 (0%)	0 (0%)	1 (0.8%)	0 (0%)	0 (0%)
Intentional Self-infliction	0 (0%)	1 (2.6%)	1 (3.3%)	4 (6.6%)	1 (1.8%)	2 (2.6%)	1 (1%)	8 (6.8%)	3 (2.3%)	3 (4.9%)
Other/Undetermined	3 (11.1%)	8 (21.1%)	5 (16.7%)	4 (6.6%)	2 (3.6%)	3 (3.9%)	9 (8.7%)	11 (9.4%)	3 (2.3%)	1 (1.6%)

### 3.2 Time series analysis

#### 3.2.1 COVID-19 time series

**Figure 1:**
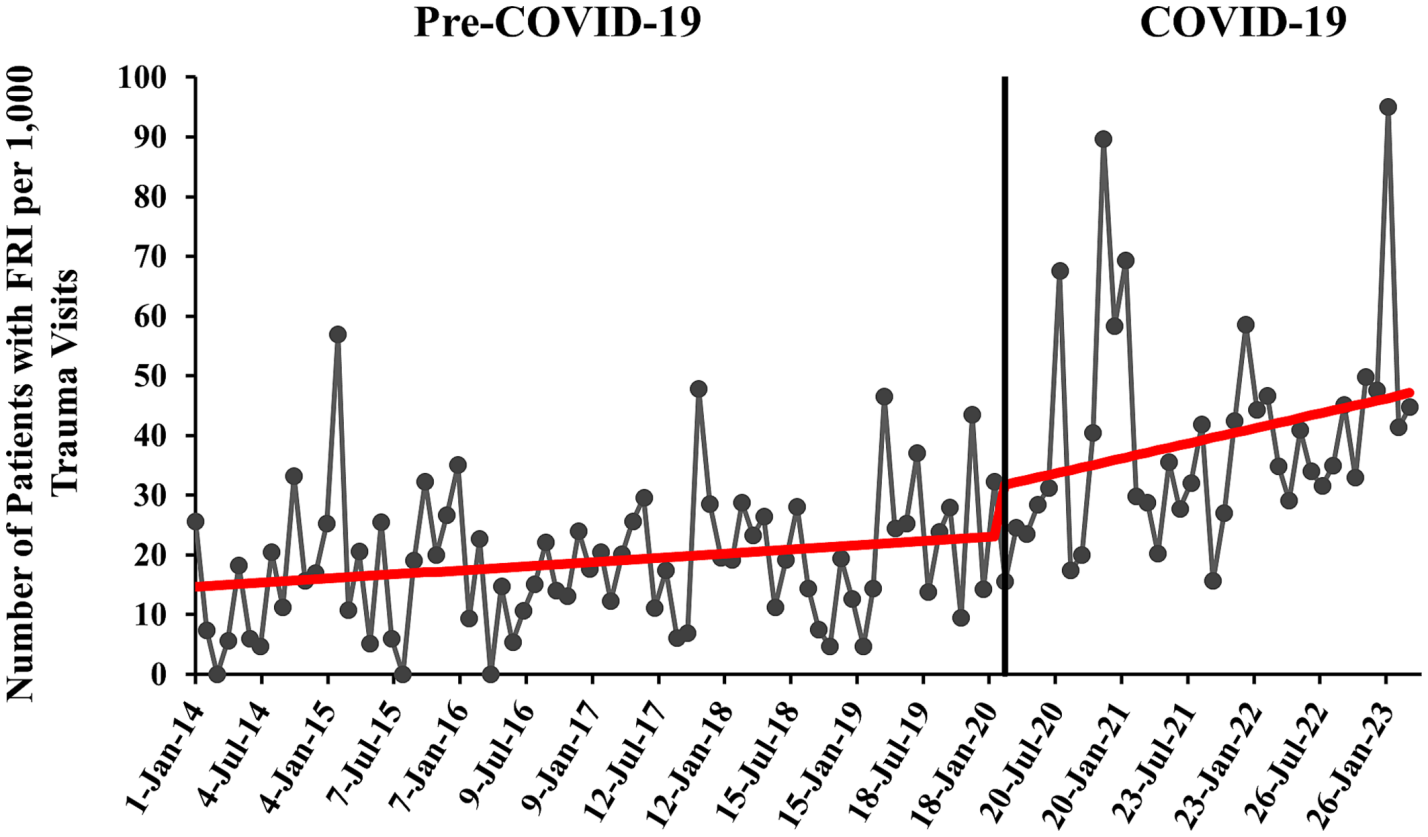
Monthly rate of pediatric patients with firearm-related injury (FRI) pre- versus during COVID-19 with overlaid interrupted time series (ITS) trend (red line). Rate of patients with FRI is calculated per 1,000 patient visits to our EDs that were qualified for and were registered in the trauma registry (trauma visits).

A baseline level of 14.5 firearm patients per 1,000 trauma visits (95% CI 8.18–20.91, *p* < 0.001) was observed pre-COVID-19 (January 2014 to February 2020) (Figure 1). The increase in rate of FRI pre-COVID-19 was not significant (β = 0.12, 95% CI -0.03-0.26, *p* = 0.130). At the start of COVID-19, there was an increase in FRI rate by 8.3 patients per 1,000 trauma visits, although this level change was not statistically significant (95% CI -2.65-19.2, *p* = 0.140). Rates of FRI during COVID-19 increased significantly by 0.42 patients per 1,000 trauma visits each month (95% CI 0.02–0.82, *p* = 0.042), although this slope did not significantly differ compared to the pre-COVID-19 slope (β = 0.12 vs. 0.42, 95% CI -0.13-0.73, *p* = 0.171). During the pandemic, the predicted number of patients with FRI per 1,000 trauma visits increased from approximately 32 in March 2020 to nearly 47 in March 2023. For additional information, please see Supplementary Table S1.

#### 3.2.2 SB 319 time series

**Figure 2:**
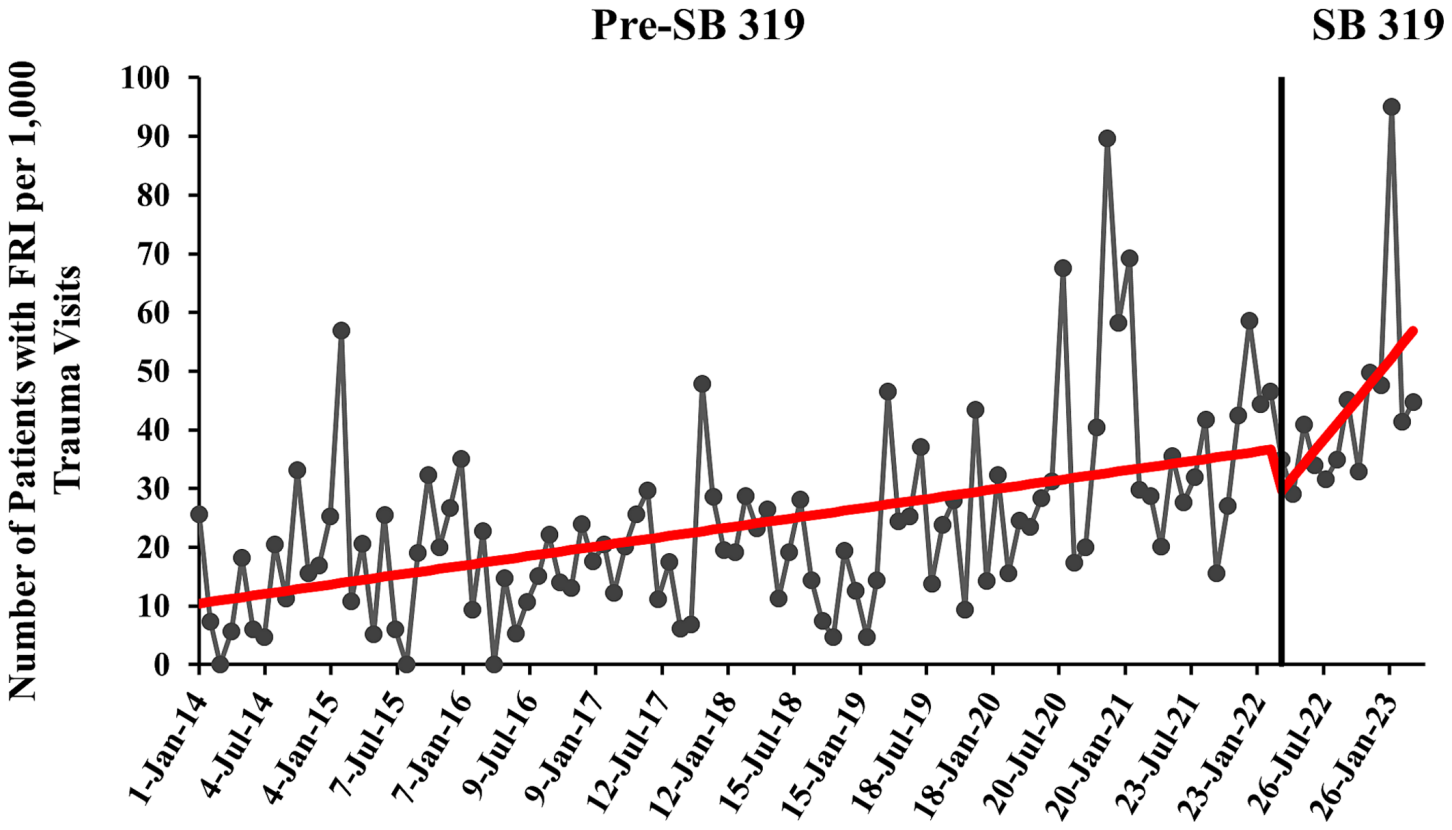
Monthly rate of pediatric patients with firearm-related injury (FRI) pre- and post-Constitutional Carry Act (SB 319) with overlaid interrupted time series (ITS) trend (red line). Rate of patients with FRI is calculated per 1,000 patient visits to our EDs that were qualified for and were registered in the trauma registry (trauma visits).

The pre-SB 319 era (January 2014 to March 2022) had an initial baseline of approximately 10 firearm patients per 1,000 trauma visits (95% CI 4.60–15.75, *p* = 0.001) (Figure 2). Pre-SB 319, FRI increased significantly at a monthly rate of 0.27 patients per 1,000 trauma visits (95% CI 0.17–0.36, *p* < 0.001). In the post-SB 319 era, FRI increased significantly by 2.3 patients per 1,000 trauma visits each month (95% CI 0.23–4.31, *p* = 0.029). One month after SB 319 was in effect, there were nearly 32 predicted patients with FRI per 1,000 trauma visits. One year after SB 319 was in effect, this prediction rose to 57 patients with FRI per 1,000 trauma visits. The difference in slopes between the pre- and post-SB 319 was 2.00, nearing significance (β = 0.26 vs. 2.26, 95% CI -0.04-4.05, *p* = 0.058). See Supplementary Table S2 for additional information.

#### 3.2.3 Combined COVID-19 and SB 319 time series

**Figure 3:**
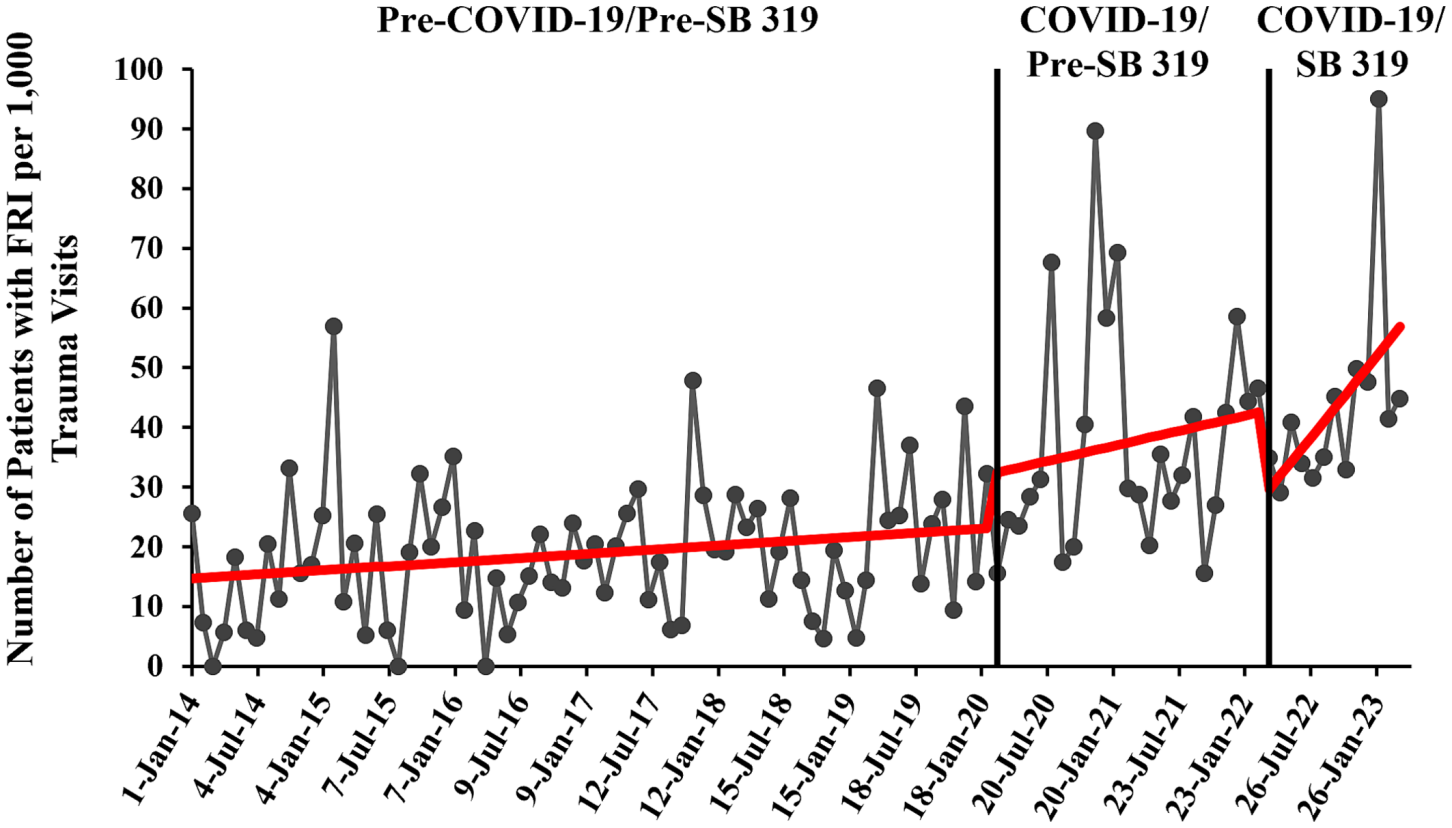
Monthly rate of pediatric patients with firearm-related injury (FRI) pre- and during COVID-19 and pre- and post-Constitutional Carry Act (SB 319) with overlaid interrupted time series (ITS) trend (red line). Rate of patients with FRI is calculated per 1,000 patient visits to our EDs that were qualified for and were registered in the trauma registry (trauma visits).

Consistent with the COVID-19 individual analysis, the time series started with an initial monthly rate of 14.5 patients with FRI per 1,000 trauma visits (95% CI 8.22–20.86, *p* < 0.001) (Figure 3). During COVID-19 and before SB 319 (March 2020–March 2022), the trend in monthly FRI rate was not significant (β = 0.42, 95% CI -0.33-1.17, *p* = 0.270). After SB 319 was put into effect, there was the previously documented significant, sustained increase of 2.3 additional patients with FRI per 1,000 trauma visits each month (95% CI 0.28–4.26, *p* = 0.026). This slope did not statistically differ with the pre-SB 319 slope (β = 0.42 vs. 2.26, 95% CI -0.28-3.98, *p* = 0.092). See Supplementary Table S3 for more information.

#### 3.2.4 Relationship between patient race and FRI outcomes

Sex, ethnicity, age, insurance status, and mechanism of injury correlated with Black and White race (*p* < 0.05) (Table 2). Black patients were more publicly insured compared to White patients (86.4% vs. 72.5%), and less privately insured (8.2% vs. 19.1%). Black patients suffered more assault injuries (49.1% vs. 23.7%), less unintentional injuries (40.9% vs. 61.8%), and less intentional self-inflicted injuries than White patients (2.7% vs. 6.1%). A larger percentage of Black patients were in the youngest cohort (0–6 years-old) compared to White patients (30.5% vs. 19.8%).

**Table 2:** Participant characteristics by race (Black versus White, *N* = 656).

Characteristic, Raw *N* (col. %) or Median (IQR)	Black Race, *N* = 525	White Race, *N* = 131	*p*-value	Unweighted SMD^a^	IPTW SMD^b,c^
Sex					
Female	132 (25.1%)	22 (16.8%)	**0.044**	0.206	0.053
Male	393 (74.9%)	109 (83.2%)			
Ethnicity					
Hispanic/Latino	4 (0.8%)	21 (16%)	**<0.001**	0.573	0.206
Non-Hispanic/Latino	521 (99.2%)	110 (84%)			
Age					
0–6	160 (30.5%)	26 (19.8%)	**0.004**	0.359	0.189
7–12	158 (30.1%)	61 (46.6%)			
13–15	161 (30.7%)	34 (26%)			
16–20	46 (8.7%)	10 (7.6%)			
Insurance, *N* = 652					
Private	43 (8.2%)	25 (19.1%)	**0.001**	0.357	0.105
Public	450 (86.4%)	95 (72.5%)			
Uninsured	28 (5.4%)	11 (8.4%)			
Mechanism of injury					
Unintentional	215 (40.9%)	81 (61.8%)	**<0.001**	0.570	0.213
Assault	258 (49.1%)	31 (23.7%)			
Child Abuse/Legal intervention	2 (0.4%)	0 (0%)			
Intentional Self-infliction	14 (2.7%)	8 (6.1%)			
Other/Undetermined	36 (6.9%)	11 (8.4%)			
ISS, *N* = 641	5 (1, 13)	4 (1, 10)	0.078	0.120	0.067

a SMDs < 0.25 are considered balanced.

b IPTW SMDs are calculated using stabilized ATE IPTW, truncated at 1 and 99%.

c Unweighted and IPTW weighted SMD are 0.417 and 0.089 for year, respectively.Bold values indicate significance at the 0.05 level.

Differences in unweighted versus weighted models over COVID-19 and SB 319 were not found, and for the sake of brevity, will not be reported. Mortality rate in the overall sample is 5.4%. Race was not found to affect mortality odds (OR 1.46, 95% CI 0.68–3.16, *p* = 0.334) (Table 3). Race was also not found to affect admission odds (OR 0.83, 95% CI 0.53–1.30, *p* = 0.427) (Supplementary Table S4). Likewise, race did not affect odds of being discharged to rehabilitation (OR 0.76, 95% CI 0.36–1.58, *p* = 0.459) (Supplementary Table S5).

**Table 3:** Unweighted and weighted mortality models, using Firth Logistic Regression *N* = 656.

Characteristic	Alive, *N* = 621 Raw *N* (row %)	Deceased, *N* = 35 Raw *N* (row %)	Unweighted OR (95% CI)	*p*-value	IPTW OR (95% CI)^a^	*p*-value
Race						
Black	499 (95%)	26 (5%)	Reference	0.334	Reference	0.266
White	122 (93.1%)	9 (6.9%)	1.46 (0.68, 3.16)		1.61 (0.70, 3.75)	
Pre-COVID-19, *N* = 281						
Race						
Black	196 (95.6%)	9 (4.4%)	Reference	0.223	Reference	0.583
White	70 (92.1%)	6 (7.9%)	1.91 (0.68, 5.39)		1.47 (0.37, 5.82)	
COVID-19, *N* = 375						
Race						
Black	303 (94.7%)	17 (5.3%)	Reference	0.812	Reference	0.244
White	52 (94.6%)	3 (5.4%)	1.16 (0.35, 3.82)		1.85 (0.66, 5.21)	
Pre-SB 319, *N* = 523						
Race						
Black	390 (94.7%)	22 (5.3%)	Reference	0.398	Reference	0.635
White	103 (92.8%)	8 (7.2%)	1.43 (0.63, 3.24)		1.27 (0.48, 3.36)	
SB 319, *N* = 133						
Race						
Black	109 (96.5%)	4 (3.5%)	Reference	0.528	Reference	0.081
White	19 (95%)	1 (5%)	1.87 (0.27, 13.1)		4.44 (0.83, 23.7)	

a IPTW weights are calculated using GBM with *N* = 10,000 trees, stabilized and trimmed at 1 and 99%; Weights adjust for sex, ethnicity, age, insurance, mechanism of injury, ISS, and year as confounding covariates.

#### 3.2.5 Relationship between insurance and mortality

Majority of patients had public insurance (82.6%), followed by private (10%), and self-pay (6.8%). Race (*p* < 0.001) and ethnicity (*p* = 0.04) associated with patient insurance (Table 4). Private insurance had the largest proportion of White patients (35.7% vs. 23.4% uninsured vs. 16.6% public). Privately insured patients had the highest mortality rate (10%), followed by uninsured patients (8.3%), and publicly insured patients (4.7%) (Table 5). In the unweighted model, publicly insured patients had 58% lower odds of death than privately insured patients (OR 0.42, 95% CI 0.18–0.99, p = 0.04). When controlled for age, sex, race, ethnicity, mechanism of injury, ISS, and year of injury, differences between mortality rates were not found (OR 0.76, 95% CI 0.23–2.53, *p* = 0.65). The remaining temporal analysis is exploratory as cell counts are less than 5. Privately insured patients had a lower mortality rate during COVID-19, not statistically different from that of publicly insured patients. Uninsured patients had a higher mortality rate during COVID-19, statistically different from that of privately insured patients.

**Table 4:** Participant characteristics by insurance (Private versus Public versus Uninsured, *N* = 697)^d^.

Characteristic, Raw *N* (col. %) or Median (IQR)	Private, *N* = 70	Public, *N* = 579	Uninsured, *N* = 48	*p*-value	Unweighted SMD^a^	IPTW SMD^b,c^
Sex						
Female	17 (24.3%)	135 (23.3%)	10 (20.8%)	0.905	0.055	0.072
Male	53 (75.7%)	444 (76.7%)	38 (79.2%)			
Race, *N* = 690						
Black	43 (61.4%)	450 (78.5%)	28 (59.6%)	**<0.001**	0.480	0.080
White	25 (35.7%)	95 (16.6%)	11 (23.4%)			
Mixed/Other Race	2 (2.9%)	28 (4.9%)	8 (17%)			
Ethnicity, *N* = 696						
Hispanic/Latino	1 (1.4%)	40 (6.9%)	6 (12.5%)	**0.048**	0.304	0.183
Non-Hispanic/Latino	69 (98.6%)	538 (93.1%)	42 (87.5%)			
Age						
0–6	14 (20%)	175 (30.2%)	8 (16.7%)	0.250	0.261	0.211
7–12	26 (37.1%)	192 (33.2%)	17 (35.4%)			
13–15	22 (31.4%)	164 (28.3%)	19 (39.6%)			
16–20	8 (11.4%)	48 (8.3%)	4 (8.3%)			
Mechanism of injury						
Unintentional	35 (50%)	261 (45.1%)	23 (47.9%)	0.087	0.314	0.179
Assault	23 (32.9%)	264 (45.6%)	19 (39.6%)			
Child Abuse/Legal Intervention	0 (0%)	2 (0.4%)	0 (0%)			
Intentional Self-infliction	7 (10%)	15 (2.6%)	1 (2.1%)			
Other/Undetermined	5 (7.1%)	37 (6.4%)	5 (10.4%)			
ISS, *N* = 679	5 (1, 17)	5 (1, 10)	4 (1, 9)	0.341	0.163	0.062

a SMDs < 0.25 are considered balanced.

b IPTW SMDs are calculated using stabilized ATE IPTW, truncated at 1 and 99%.

c Unweighted and IPTW weighted SMD are 1.023 and 0.788 for year, respectively.

d Four patients who had unknown insurance (*N* = 3) and other insurance (*N* = 1) were excluded.Bold values indicate significance at the 0.05 level.

**Table 5:** Unweighted and weighted mortality regression models, using Firth Logistic Regression *N* = 697.

Characteristic	Alive, *N* = 659 Raw *N* (row %)	Deceased, *N* = 38 Raw *N* (row %)	Unweighted OR (95% CI)	*p*-value	IPTW OR (95% CI)^a,b^	*p*-value
Insurance^c^						
Private	63 (90%)	7 (10%)	Reference		Reference	
Public	552 (95.3%)	27 (4.7%)	0.42 (0.18, 0.99)	**0.047**	0.76 (0.23, 2.53)	0.652
Uninsured	44 (91.7%)	4 (8.3%)	0.86 (0.25, 2.96)	0.806	2.08 (0.37, 11.6)	0.404
Pre-COVID-19, *N* = 298						
Insurance						
Private	35 (87.5%)	5 (12.5%)	Reference		Reference	
Public	208 (95.4%)	10 (4.6%)	0.33 (0.11, 0.98)	**0.046**	0.67 (0.13, 3.54)	0.640
Uninsured	39 (97.5%)	1 (2.5%)	0.25 (0.04, 1.61)	0.144	0.60 (0.04, 10.3)	0.726
COVID-19, *N* = 399						
Insurance						
Private	28 (93.3%)	2 (6.7%)	Reference		Reference	
Public	344 (95.3%)	17 (4.7%)	0.58 (0.14, 2.35)	0.714	0.68 (0.14, 3.33)	0.639
Uninsured	5 (62.5%)	3 (37.5%)	7.26 (1.05, 50.3)	**0.045**	6.67 (0.73, 60.8)	0.093
Pre-SB 319, *N* = 552						
Insurance						
Private	58 (89.2%)	7 (10.8%)	Reference		Reference	
Public	418 (94.8%)	23 (5.2%)	0.44 (0.18, 1.05)	0.063	0.74 (0.22, 2.52)	0.629
Uninsured	43 (93.5%)	3 (6.5%)	0.63 (0.16, 2.40)	0.496	1.30 (0.18, 9.43)	0.795
SB 319, *N* = 145						
Insurance						
Private	5 (100%)	0 (0%)	NA		NA	
Public	134 (97.1%)	4 (2.9%)	Reference		Reference	
Uninsured	1 (50%)	1 (50%)	0.03 (0.002, 0.63)	**0.023**	0.05 (0.004, 0.68)	**0.024**

a IPTW weights are calculated using GBM with *N* = 10,000 trees, stabilized and trimmed at 1 and 99%; Weights adjust for sex, race, ethnicity, age, mechanism of injury, ISS, and year as confounding covariates.

b IPTW weighted models additionally include year as a continuous covariate due to post-weight SMD imbalance (Weighted SMD = 0.788).

^c^Four patients who had unknown insurance (*N* = 3) and other insurance (*N* = 1) were excluded.Bold values indicate significance at the 0.05 level.

## 4 Discussion

Our study highlights an alarming rise in pediatric FRI in pediatric level I and level II trauma centers in Atlanta, Georgia over the last 9 years. Results revealed distinctions in the rates of FRI during two notable dates, the start of the COVID-19 pandemic and the effective date of state firearm law SB 319. These findings are consistent with other studies documenting similar increases over time, some in relation to the start of COVID-19 (9, 10, 13, 40) and others not (1, 18). In the present study, and consistent with past literature, patients in our sample were majority male, Black, publicly insured, and injured by assault and unintentional shootings (19). Our sample was younger in age compared to national samples, where a majority of pediatric patients with FRI are in the older adolescent age group (19, 41). This is due, in-part, to our trauma center’s proximity to adult trauma centers; patients 15 years-old and older are often routed to adult centers.

Before the COVID-19 pandemic, a significant trend in pediatric firearm rate was not found. During COVID-19, there was a sustained increase of an additional 0.42 pediatric patients with FRI per 1,000 trauma visits each month. This increase, however, was not statistically different from pre-COVID-19. Moreover, when the COVID-19 period was separated into pre- and post-SB 319, the COVID-19/Pre-SB 319 slope was insignificant. We have two possible hypotheses for these findings. First, this may suggest that the increase in FRI rates during COVID-19 was driven by the introduction of SB 319 in our state. Alternatively, prior studies that observed increased FRI rates during COVID-19 analyzed a shorter period of time, typically 6-months to 1.5-years after the start of COVID-19 (9, 10, 13, 40). Increased rates of FRI were associated with acute changes during the start of the pandemic, such as school closures and record-setting firearm purchases (9, 10, 13). Today, there are arguably less societal and economic disruptions resulting from the pandemic.

A unique contribution of this manuscript was the inclusion of The Constitutional Carry Act, a Georgia law effective since April 2022 that allows for concealed carry of firearms in public without a permit. A recent study in West Virginia found that after a concealed firearm carry law was enacted, monthly firearm mortalities in the state increased, along with brief spikes in firearm sales and homicide (42). This suggests that concealed carry laws directly impact how people purchase and use guns and may lead to increases in firearm-related assaults. In our study, there was a sustained increase in pediatric FRI by 2.3 additional patients per 1,000 trauma visits each month after the law was in effect. Given the restricted timeline (12-months post-law) and nature of the study assessing one hospital system, outliers may be exaggerating this association. Over time, we will be able to assess the association of the law and pediatric FRI more accurately. However, in the year after the law was in effect, even the lowest predicted rates of pediatric FRI are remarkably high, unlike in previous periods. This concerning trend necessitates intervention and continued evaluation of incidence rates.

Studies have found that among the U.S., Southern states have the highest rates of pediatric FRI incidence (19), have worsening pediatric FRI mortality rates (1), and have one of the highest rates of pediatric firearm homicide (16). It has been estimated that 49% of Georgia households own a firearm (43). Southern states have high rates of firearm ownership and unsafe storage (44), as defined by storing firearms loaded and unlocked. Households where children and adolescents suffered unintentional injuries or intentionally self-inflicted injuries are more likely to have unlocked, loaded firearms in the home that were stored with ammunition (45). One study found that Black households are more likely than White households to store firearms loaded and unlocked (44). Almost half of the children and adolescents injured by firearms in our sample were injured unintentionally. This often looks like a child getting ahold of an unlocked, loaded firearm at home, playing with it, and accidentally injuring themselves or another child (16, 45, 46). Consistent with the literature, it is possible that many of the pediatric FRI in our sample occurred due to the availability of firearms, and non-powder guns, within the household.

Although we did not find statistical differences in mortality odds between racial groups, there were four times as many Black patients with FRI than there were White patients in our sample. The disproportionate increase in Black patients seen over time is corroborated in other studies as well (19), although our cohort appears to have more Black patients than national studies (74.9% vs. 50%) (1, 19). This may be due in-part to the racial profile of Atlanta. However, further review of the trauma registry data did not show similar racial distributions nor differences in rates of trauma over time, suggesting a discrepancy in rates of FRI in Black children and adolescents compared to White. Studies have found that deprived (21) or low-income areas correlate with higher pediatric FRI risk (19) and mortality rates (1). Studies have shown that Black and other minoritized race groups come from low socio-economic backgrounds that put them at increased risk for injuries (26). Future studies should investigate the correlation between neighborhood-level SDH, race, and FRI in Atlanta.

We did not find differences in acute outcomes based on race. A national analysis of pediatric FRI mortality data found that Black patients suffer 4 times the mortality rate of White patients (34), while another looking at national hospitalizations found that Black patients had lower odds of mortality than White patients (41). These mortality disparities often relate to mechanism of injury; a recent study found that White patients had higher odds of mortality due to higher rates of intentional self-inflicted injuries (32). Concurrent with national trends, Black patients in our sample suffered more assault injuries and less unintentional and intentional self-inflicted injuries than White patients did (32, 41, 47). The uneven distribution of race in our sample may have contributed to our lack of findings in mortality disparities. In addition, mortality was difficult to model as it was a rare outcome. The study sample’s unique characteristics may have contributed as well, such as the large proportion of younger children and adolescents.

Patients with private insurance had significantly higher odds of mortality than patients with public insurance. However, when controlled for confounding factors such as race, year of injury, and mechanism of injury, this difference was not found. As this analysis was more exploratory in nature, further investigation is needed to understand what is driving this change. Privately insured patients suffered more intentional self-inflicted injuries compared to publicly insured patients (10% vs. 2.6%); this mechanism is known to be more severe and lethal than other mechanisms and may help to explain differences in mortality between these groups (41). Other FRI studies that examined insurance have found disparities in mortality between uninsured and insured patients (19, 23). To our knowledge, we are among few studies that found disparities between private and public insurance, particularly in pediatric FRI. One pediatric trauma study found that insurance, rather than race, predicted mortality (25); another found that a combination of both is necessary to understand disparities (24). The disparities found in the present study are likely better explained by SDH; future investigations are needed to understand the intricacies between race, insurance, other SDH, and acute outcomes following FRI. Notably, insurance is an important SDH; it can serve as a proxy to socioeconomic status (SES), and it affects hospital-based care and care-seeking behaviors (39). However, it mainly reflects individual-level factors. Future studies will benefit from examining neighborhood-level SDH, such as neighborhood depravity. Additionally, mortality rates in our sample were found to have fluctuated across COVID-19 in privately insured patients but not publicly insured patients. This suggests a need for continued investigation into temporal differences.

There are several limitations within our study that are worth noting. Race and insurance analysis were balanced by mechanism of injury, among other factors, to avoid its potential confounding effects with the outcome. As such, the effects of mechanism of injury could not be included as a predictor in the regression analysis but should be addressed in future studies. As an outcome, mortality was a rare occurrence and presents challenges for constructing and interpreting models. Older adolescents are often sent to a neighboring adult Level I trauma center in Atlanta; thus, our older adolescent group is likely smaller and less representative of the adolescent population with FRI in our area. This study is also limited to available trauma registry data, which only collects acute outcomes, rather than long-term outcomes. Patients who were not at our hospitals (i.e., patients that passed away at the scene) are not included in this study. Notably, we did not validate trauma registry data with review of electronic medical records. Mechanism of injury, which is taken from ICD E-code, could be inaccurate (48). There may also be an overrepresentation of Medicaid insurance in the Trauma registry, as our institutional trauma registry defaults insurance type to Medicaid if the field is not entered. Additionally, race and ethnicity are pulled from medical records, which are entered by hospital staff who register patients upon entry. There is a possibility that given the busy nature of the hospital and severity of these injuries that some of the race and ethnicity fields could have been assumed based on phenotype rather than inquired of the patient or their family. Finally, our study was limited to single center design and may not be representative of the rest of our state or national pediatric rates of FRI. There may be a chance our hospital systems are receiving more of the state’s proportion of pediatric patients with FRI over time, this can be assessed in a future study examining state-wide pediatric FRI.

## 5 Conclusion

The observed increase of FRI in children and adolescents at our trauma centers is highly concerning. Both COVID-19 and the Constitutional Carry Act (SB 319) were followed by significant sustained increases in pediatric patients with FRI. The continual increase of children and adolescents injured by firearms requires both evidence-based interventions and continued research to evaluate trends and inform interventions. The high concentration of Georgian children and adolescents injured necessitates interventions in the Atlanta and surrounding area, such as violence prevention programs and safe firearm storage training (49). Further investigation into SDH and pediatric FRI are needed to understand the observed insurance-based differences in mortality odds and to identify factors driving the increased FRI incidence among Black children and adolescents. Although racial disparities were not found in acute outcomes, majority of pediatric patients survive FRI, thus necessitating research into long-term outcomes, including long-term physical, cognitive, and emotional functioning. With further research into FRI, acute and long-term outcomes, disparities, and the intersection of SDH, we can address the increase in pediatric FRI at its source and help children and adolescents live high-quality lives.

## Data availability statement

The datasets presented in this article are not readily available due to concerns regarding patient confidentiality. Requests to access the datasets should be directed to the corresponding author.

## Ethics statement

The studies involving humans were approved by Children's Healthcare of Atlanta Institutional Review Board. The studies were conducted in accordance with the local legislation and institutional requirements. The ethics committee/institutional review board waived the requirement of written informed consent for participation from the participants or the participants’ legal guardians because this study is a retrospective chart review and thus consent was not required.

## Author contributions

MM: Conceptualization, Data curation, Investigation, Writing – original draft, Writing – review & editing. GB: Conceptualization, Writing – original draft. KP: Writing – original draft. SG: Formal analysis, Methodology, Visualization, Writing – review & editing, Software. LJ: Formal analysis, Methodology, Visualization, Writing – review & editing, Software. KD: Writing – review & editing. AR: Project administration, Resources, Writing – review & editing. LB: Conceptualization, Investigation, Project administration, Supervision, Writing – original draft, Writing – review & editing.
