# Supplementary material for: Trends in pediatric firearm-related injuries and disparities in acute outcomes
Source: Front Public Health. 2024 Mar 19;12:1339394. doi: 10.3389/fpubh.2024.1339394 (PMC10985139; doi:10.3389/fpubh.2024.1339394)
Supplement: Supplementary file 1 [file Table_1.docx]

Supplementary Materials

**Supplementary Table 1.** Interrupted time series (ITS) estimates, 95% CI, and p-values for monthly firearm-related injuries pre- and during COVID-19.

| Parameter | Estimate (95% CI)^1^ | *p*-value |
| --- | --- | --- |
| Intercept | 14.544 (8.179-20.908) | **<0.001*** |
| Pre-COVID-19 Slope | 0.115 (-0.033-0.262) | 0.130 |
| Level Change | 8.277 (-2.646-19.2) | 0.140 |
| COVID-19 Slope | 0.416 (0.015-0.816) | **0.042*** |
| Difference in Slopes | 0.301 (-0.127-0.728) | 0.171 |

^1^Estimates calculated using segmented linear regression with OLS and 0 lags (i.e., no autocorrelation was present in the data).

*Bold values indicate significance at the 0.05 level.
