# Supplementary material for: Trends in pediatric firearm-related injuries and disparities in acute outcomes
Source: Front Public Health. 2024 Mar 19;12:1339394. doi: 10.3389/fpubh.2024.1339394 (PMC10985139; doi:10.3389/fpubh.2024.1339394)
Supplement: Supplementary file 2 [file Table_2.docx]

Supplementary Materials

**Supplementary Table 2.** Interrupted time series (ITS) estimates, 95% CI, and p-values for monthly firearm-related injuries pre- and post-Constitutional Carry Act (SB 319).

| Parameter | Estimate (95% CI)^1^ | *p*-value |
| --- | --- | --- |
| Intercept | 10.175 (4.596-15.754) | **0.001*** |
| Pre-SB 319 Slope | 0.267 (0.171-0.364) | **<0.001*** |
| Level Change | -9.306 (-26.418-7.806) | 0.289 |
| SB 319 Slope | 2.269 (0.227-4.311) | **0.029*** |
| Difference in Slopes | 2.002 (-0.042-4.046) | 0.058 |

^1^Estimates calculated using segmented linear regression with OLS and 0 lags (i.e., no autocorrelation was present in the data).

*Bold values indicate significance at the 0.05 level.
