# Supplementary material for: Trends in pediatric firearm-related injuries and disparities in acute outcomes
Source: Front Public Health. 2024 Mar 19;12:1339394. doi: 10.3389/fpubh.2024.1339394 (PMC10985139; doi:10.3389/fpubh.2024.1339394)
Supplement: Supplementary file 3 [file Table_3.docx]

Supplementary Materials

**Supplementary Table 3.** Interrupted time series (ITS) estimates, 95% CI, and p-values for monthly firearm-related injuries pre- and during COVID-19 and pre- and post- Constitutional Carry Act (SB 319).

| Parameter | Estimate (95% CI)^1^ | *p*-value |
| --- | --- | --- |
| Intercept | 14.544 (8.224, 20.863) | **<0.001*** |
| Pre-COVID-19/Pre-SB 319 Slope (1) | 0.115 (-0.032, 0.261) | 0.127 |
| Level Change 1 | 8.945 (-3.761, 21.650) | 0.171 |
| COVID-19/Pre-SB 319 Slope (2) | 0.420 (-0.327, 1.166) | 0.270 |
| Difference in (1) v. (2) slopes | 0.305 (-0.456, 1.065) | 0.434 |
| Level Change 2 | -15.134 (-34.102, 3.834) | 0.121 |
| COVID-19/SB 319 Slope (3) | 2.269 (0.275, 4.264) | **0.026*** |
| Difference in (2) v. (3) slopes | 1.850 (-0.280, 3.979) | 0.092 |

^1^Estimates calculated using segmented linear regression with OLS and 0 lags (i.e., no autocorrelation was present in the data).

*Bold values indicate significance at the 0.05 level.
