# Supplementary material for: Trends in pediatric firearm-related injuries and disparities in acute outcomes
Source: Front Public Health. 2024 Mar 19;12:1339394. doi: 10.3389/fpubh.2024.1339394 (PMC10985139; doi:10.3389/fpubh.2024.1339394)
Supplement: Supplementary file 4 [file Table_4.docx]

Supplementary Materials

**Supplementary Table 4.** Of Black or White patients, unweighted and weighted admission to the hospital from ED (ICU, OR, floor, or direct admit) versus discharge home from the ED, using Firth Logistic Regression N=639.

| Characteristic | Home, N=152  Raw N (row %) | Admitted, N=487  Raw N (row %) | Unweighted  OR (95% CI) | *p*-value | IPTW  OR (95% CI)^1^ | *p*-value |
| --- | --- | --- | --- | --- | --- | --- |
| Race^2^ |  |  |  |  |  |  |
| Black | 119 (23.2%) | 395 (76.8%) | Reference | 0.427 | Reference | 0.994 |
| White | 33 (26.4%) | 92 (73.6%) | 0.83 (0.53, 1.30) |  | 1.00 (0.59, 1.71) |  |
| Pre-COVID-19, N=272 |  |  |  |  |  |  |
| Race |  |  |  |  |  |  |
| Black | 39 (19.5%) | 161 (80.5%) | Reference | 0.774 | Reference | 0.802 |
| White | 15 (20.8%) | 57 (79.2%) | 0.91 (0.47, 1.76) |  | 1.12 (0.47, 2.67) |  |
| COVID-19, N=367 |  |  |  |  |  |  |
| Race |  |  |  |  |  |  |
| Black | 80 (25.5%) | 234 (74.5%) | Reference | 0.188 | Reference | 0.740 |
| White | 18 (34%) | 35 (66%) | 0.66 (0.35, 1.23) |  | 0.89 (0.45, 1.76) |  |
| Pre-SB 319, N=508 |  |  |  |  |  |  |
| Race |  |  |  |  |  |  |
| Black | 85 (21.1%) | 317 (78.9%) | Reference | 0.562 | Reference | 0.857 |
| White | 25 (23.6%) | 81 (76.4%) | 0.86 (0.52, 1.43) |  | 0.95 (0.52, 1.72) |  |
| SB 319, N=131 |  |  |  |  |  |  |
| Race |  |  |  |  |  |  |
| Black | 34 (30.4%) | 78 (69.6%) | Reference | 0.305 | Reference | 0.885 |
| White | 8 (42.1%) | 11 (57.9%) | 0.59 (0.22, 1.61) |  | 1.09 (0.34, 3.51) |  |

^1^IPTW weights are calculated using GBM with N=10,000 trees, stabilized and trimmed at 1% and 99%; Weights adjust for sex, ethnicity, age, insurance, mechanism of injury, ISS, and year as confounding covariates.

^2^17 patients that identified as Black or White race were excluded: n=9 that died in the ED and n=8 that were transferred to another hospital from the ED.
