# Supplementary material for: Trends in pediatric firearm-related injuries and disparities in acute outcomes
Source: Front Public Health. 2024 Mar 19;12:1339394. doi: 10.3389/fpubh.2024.1339394 (PMC10985139; doi:10.3389/fpubh.2024.1339394)
Supplement: Supplementary file 5 [file Table_5.docx]

Supplementary Materials

**Supplementary Table 5.** Of Black or White admitted patients, unweighted and weighted discharge home from the hospital versus discharge to inpatient rehabilitation from the hospital, using Firth Logistic Regression N=445.

| Characteristic | Home, N=381  Raw N (row %) | Rehab, N=64  Raw N (row %) | Unweighted  OR (95% CI) | *p*-value | IPTW  OR (95% CI)^1^ | *p*-value |
| --- | --- | --- | --- | --- | --- | --- |
| Race^2^ |  |  |  |  |  |  |
| Black | 311 (85%) | 55 (15%) | Reference | 0.459 | Reference | 0.460 |
| White | 70 (88.6%) | 9 (11.4%) | 0.76 (0.36, 1.58) |  | 0.72 (0.31, 1.71) |  |
| Pre-COVID-19, N=199 |  |  |  |  |  |  |
| Race |  |  |  |  |  |  |
| Black | 126 (83.4%) | 25 (16.6%) | Reference | 0.811 | Reference | 0.708 |
| White | 41 (85.4%) | 7 (14.6%) | 0.90 (0.37, 2.19) |  | 0.81 (0.26, 2.47) |  |
| COVID-19, N=246 |  |  |  |  |  |  |
| Race |  |  |  |  |  |  |
| Black | 185 (86.1%) | 30 (13.9%) | Reference | 0.342 | Reference | 0.558 |
| White | 29 (93.5%) | 2 (6.5%) | 0.52 (0.13, 2.02) |  | 0.68 (0.19, 2.47) |  |
| Pre-SB 319, N=362 |  |  |  |  |  |  |
| Race |  |  |  |  |  |  |
| Black | 246 (84.3%) | 46 (15.7%) | Reference | 0.605 | Reference | 0.636 |
| White | 61 (87.1%) | 9 (12.9%) | 0.82 (0.38, 1.75) |  | 0.81 (0.34, 1.95) |  |
| SB 319, N=83 |  |  |  |  |  |  |
| Race |  |  |  |  |  |  |
| Black | 65 (87.8%) | 9 (12.2%) | Reference | 0.518 | Reference | 0.564 |
| White | 9 (100%) | 0 (0%) | 0.36 (0.02, 7.86) |  | 0.40 (0.02, 8.90) |  |

^1^IPTW weights are calculated using GBM with N=10,000 trees, stabilized and trimmed at 1% and 99%; Weights adjust for sex, ethnicity, age, insurance, mechanism of injury, ISS, and year as confounding covariates.

^2^211 patients that identified as Black or White race were excluded: n=170 were not admitted to the hospital, n=26 died, n=13 discharged to another hospital/intermediate care facility, and n=2 discharged/transferred to court/law enforcement.
